# Partial order relation–based gene ontology embedding improves protein function prediction

**DOI:** 10.1093/bib/bbae077

**Published:** 2024-03-05

**Authors:** Wenjing Li, Bin Wang, Jin Dai, Yan Kou, Xiaojun Chen, Yi Pan, Shuangwei Hu, Zhenjiang Zech Xu

**Affiliations:** College of Computer Science and Software, Shenzhen University, Shenzhen, China; School of Mathematics and Computer Sciences, Nanchang University, Nanchang, China; Center for Quantum Technology Research and School of Physics, Beijing Institute of Technology, Beijing, China; Xbiome, Scientific Research Building, Tsinghua High-Tech Park, Shenzhen, China; College of Computer Science and Software, Shenzhen University, Shenzhen, China; Faculty of Computer Science and Control Engineering Shenzhen Institute of Advanced Technology, Chinese Academy of Sciences 1068 Xueyuan Avenue, Shenzhen University Town, Shenzhen, China; Xbiome, Scientific Research Building, Tsinghua High-Tech Park, Shenzhen, China; School of Mathematics and Computer Sciences, Nanchang University, Nanchang, China; State Key Laboratory of Food Science and Technology, Nanchang University, Nanchang, China

**Keywords:** Gene Ontology, protein annotation, representation learning, protein function prediction, partial order constraint

## Abstract

Protein annotation has long been a challenging task in computational biology. Gene Ontology (GO) has become one of the most popular frameworks to describe protein functions and their relationships. Prediction of a protein annotation with proper GO terms demands high-quality GO term representation learning, which aims to learn a low-dimensional dense vector representation with accompanying semantic meaning for each functional label, also known as embedding. However, existing GO term embedding methods, which mainly take into account ancestral co-occurrence information, have yet to capture the full topological information in the GO-directed acyclic graph (DAG). In this study, we propose a novel GO term representation learning method, PO2Vec, to utilize the partial order relationships to improve the GO term representations. Extensive evaluations show that PO2Vec achieves better outcomes than existing embedding methods in a variety of downstream biological tasks. Based on PO2Vec, we further developed a new protein function prediction method PO2GO, which demonstrates superior performance measured in multiple metrics and annotation specificity as well as few-shot prediction capability in the benchmarks. These results suggest that the high-quality representation of GO structure is critical for diverse biological tasks including computational protein annotation.

## INTRODUCTION

Proteins are the main bearers of life activities, and understanding their functions is important for unlocking the biological code. Experimental protein function annotation is time-consuming and extremely expensive. Next-generation sequencing technologies have resulted in a large number of protein sequences to be annotated, thus leading to an urgent need for low-cost and high-efficiency protein function annotation methods [1]. Deep learning [2] has shown promising results in predicting protein structure and protein function [3–7] over traditional methods that are based on sequence similarity and homology search. However, existing deep learning–based protein function prediction methods still confront significant challenges when dealing with a large and complex hierarchical functional label space. The first one, on the protein side, is how to extract effective feature representations from amino acid (AA) sequences. Benefiting from the development of natural language processing (NLP) techniques [8], self-supervised pre-trained protein language models based on transformer [9] have been shown to effectively encode embedding representations of AA sequences [4, 10, 11]. The second one, from the label side, is how to accurately project low-dimensional embedding of protein into a large-scale, hierarchical and extremely unbalanced functional label space for protein function prediction task [12]. Early deep learning approaches [5] directly use a flat classifier for protein function prediction and ignore the relationships between functional labels. Until recently, several studies [6, 7] started to learn a low-dimensional geometric representation (e.g. a low-dimensional dense vector) for each functional label by exploring their inter-relationships and integrating this representation learning for downstream protein annotation. For example, Zhou *et al*. [6] use a two-layer graph convolutional network (GCN) [13] while Cao and Shen [7] use simple matrix multiplication to learn the vector representation of function labels. However, the complexity of functional labels still warrants more sophisticated modeling to capture their full relationships and semantic meanings to improve protein function prediction.

Gene Ontology (GO) [14] has become one of the most popular functional label systems to characterize proteins and their relationships, including their molecular functions (molecular function ontology, MFO), their subcellular locations (cellular component ontology, CCO) and biological process in which the proteins are involved (biological process ontology, BPO). The GO terms are organized in a hierarchical DAG, with shallow terms representing broad, abstract semantics and deep terms representing concrete, precise semantics. In the protein function annotation task, each protein is usually annotated with more than one GO term, constituting a multi-label classification problem in machine learning.

In this work, we propose a novel GO term representation learning method PO2Vec to learn embedding representation for GO terms. In contrast to existing methods [15–18], which typically rely on the ancestral co-occurrence, PO2Vec learns topological information by exploring the shortest reachable path–based partial order relationships. In addition, we apply the pre-trained GO embedding to the protein function classification task and propose a new protein function prediction method, PO2GO. Extensive evaluations demonstrate that PO2Vec outperforms existing methods, both IC-based [19] and deep learning-based [15, 17, 18, 20], in learning GO embedding representations across a range of biological tasks. Additionally, PO2GO outperforms alternative methods [6, 7] for protein annotation, despite utilizing the topological structure information of GO. The novelties of this approach are 2-fold. First, PO2Vec is the first method that utilizes the important partial order relationships in GO for learning better GO term embeddings. Second, we propose a contrastive learning method to model the partial order relationships and the experimental results show that PO2Vec can better capture the topological and biological information of GO terms. Benefiting from PO2Vec’s effective representation learning, PO2GO outperforms existing protein annotation methods in terms of information content and when dealing with insufficient training samples. These results encourage further extension of our method in exploiting other types of protein-related features or in learning the representations of other ontology graphs.

## METHODS & MATERIALS

In this paper, we propose a novel protein function prediction method, Partial Order to Gene Ontology (PO2GO), as shown in Figure 2. The architecture of the new method consists of three main components: (i) protein feature extractor encodes a protein sequence into a vector; (ii) GO term encoder obtains embedding for each GO term; and (iii) joint modeling predictor performs protein function prediction by conducting GO terms embedding database searching. We will introduce these modules in the following subsections.

### Protein feature extractor

In this paper, we used the ESM-1b [4] to obtain protein embedding from AA sequences since ESM-1b is superior in terms of moderate model size and strong feature representation ability [4, 11]. ESM-1b is a pre-trained protein language model (PLM), using RoBERTa [21] architecture, trained on over 250 million protein sequences from the UniRef database [22]. Compared with RoBERTa, AA representations extracted by ESM-1b contain information about biological properties that can be directly applied to downstream protein tasks. Specifically, given a protein $p$ with $L$ AAs, we use ESM-1b to obtain the embedding of each AA and form a matrix $B\in{\mathbb{R}}^{d\times L}$ where $d$ is the AA embedding dimension. Following the study [11], we use the same mean pooling strategy to aggregate AA features and obtain the protein embedding of $p$ as $f(p)=\mathrm{mean}(B)\in{\mathbb{R}}^{d\times 1}$.

### PO2Vec for GO term embedding

**Figure 1:**
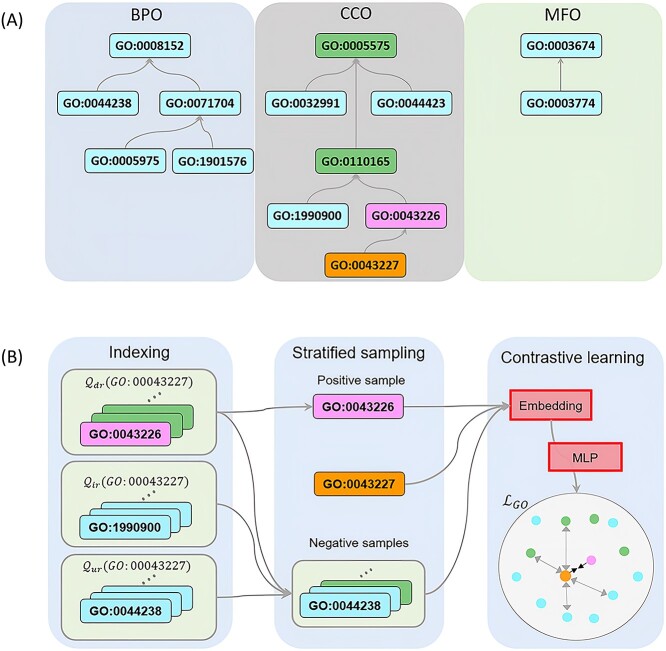
Illustration of partial order constraint used for PO2Vec embedding algorithm. (**A**) Snippets of GO hierarchy demonstrating the shortest reachable path-based partial order constraints. Given a GO term, it should be semantically more similar to its closely directly reachable term than its distantly reachable term, which include directly reachable, indirectly reachable and unreachable terms. (**B**) The flowchart of PO2Vec algorithm incorporating partial order constraint. With contrastive learning, semantically close terms are driven together while distant terms are driven apart in the embedding space.

In the protein function prediction task, our aim is to map each protein to a set of GO terms. Given a set of $m$ GO terms $\mathcal{T}=\left\{{t}_1,{t}_2,\dots, {t}_m\right\}$, we aim to learn an embedding function $e\left(\cdot \right)$ to obtain the embedding $e\left({t}_i\right)\in{\mathbb{R}}^{d^{\prime}\times 1}$ (where ${d}^{\prime }$ is the term embedding dimension) for a term ${t}_i$. There are several types of relationships between GO terms, the most common of which are is_a and part_of. Specifically, ${t}_1$ is_a ${t}_2$ means that ${t}_1$ is a subclass of ${t}_2$; ${t}_3$ part_of ${t}_4$ indicates that whenever ${t}_3$ is present, it is always a part of ${t}_4$. The relationships between GO terms could be transitive. For example, as shown in Figure 1A, if membrane-bounded organelle (GO:0043227) is_a organelle (GO:0043226) and organelle (GO:0043226) is_a cellular anatomical entity (GO:0110165), then membrane-bounded organelle (GO:0043227) is_a cellular anatomical entity (GO:0110165). This transmission relationship forms a hierarchy between terms, in which shallower terms represent more abstract semantics and deeper terms represent more concrete semantics. The hierarchy is crucial for protein function annotation. For example, if a protein is annotated as membrane-bounded organelle (GO:0043227), then it should also be annotated as organelle (GO:0043226) and cellular anatomical entity (GO:0110165); this transmission rule is known as the true path rule [23] in protein function annotation. Although a number of relationships exist between GO terms, only is_a and part_of, which account for 88% of relationships (Supplementary Table S1 available online at http://bib.oxfordjournals.org/), could safely transfer annotations [14, 24]. Since is_a and part_of are the majority of the relationships, we only consider these two relationships in our modeling for the sake of simplicity. In the following, we first define the partial order constraints to guide the embedding learning and then propose a contrastive learning method to learn the GO term embeddings.

### Partial order constraint

In GO representation learning, the majority of methods [15–18] that rely on the GO DAG structure assume that the similarity of two terms is primarily determined by the topological structure of the GO DAG, despite the fact that the similarity may also be influenced by other factors, such as the number of annotations of terms. Intuitively, $sim\left(e\left({t}_i\right),e\left({t}_j\right)\right)$, the similarity between two terms ${t}_i$ and ${t}_j$, should be related to the length of the shortest path between ${t}_i$ and ${t}_j$. For example, both organelle (GO:0043226) and cellular anatomical entity (GO:0110165) are ancestors of membrane-bounded organelle (GO:0043227) (Figure 1A). However, the semantic similarity between organelle (GO:0043226) and membrane-bounded organelle (GO:0043227) should be greater than the semantic similarity between cellular anatomical entity (GO:0110165) and membrane-bounded organelle (GO:0043227). Although most existing GO term embedding methods [15–18, 25] capture the co-occurrence relationships among a term and its ancestors, few take the path between two terms into consideration for GO term embedding learning.

To solve the above problem, we propose a new method for learning GO term embedding by taking the path between terms into consideration. Given two terms ${t}_i$ and ${t}_j$, within a GO DAG, we define the shortest reachable path (SRP), $srp\left({t}_i,{t}_j\right)$, based on the following three cases:

1. Direct reachability: ${t}_j$ is said to be directly reachable from ${t}_i$ if there exists a directed path that starts at ${t}_i$ and ends at ${t}_j$. This typically applies to node pairs with ancestral relationships. $srp\left({t}_i,{t}_j\right)$ between ${t}_i$ and ${t}_j$ is defined as the path with minimum number of edges connected ${t}_i$ and ${t}_j$. $len\left( srp\left({t}_i,{t}_j\right)\right)$ returns the number of edges, for example, $len\left( srp\left(\mathrm{GO}:0043227,\mathrm{GO}:0110165\right)\right)=2$.
2. Indirect reachability: ${t}_j$ is said to be indirectly reachable from ${t}_i$ if ${t}_j$ is not directly reachable from ${t}_i$ but there exists a term ${t}_k$ such that ${t}_k$ is directly reachable from both ${t}_i$ and ${t}_j$. This is often the case for sibling or sibling-like term pairs. The definition of $srp\left({t}_i,{t}_j\right)$ is that the path with minimum number of edges connected ${t}_i$ and ${t}_j$ among all indirect reachable paths. The length of SRP is calculated by adding 0.5 to the number of edges, for example, $len\left( srp\left(\mathrm{GO}:00043227,\mathrm{GO}:1990900\right)\right)=3.5$.
3. Unreachability: ${t}_j$ is said to be unreachable from ${t}_i$ if ${t}_j$ is neither directly nor indirectly reachable from ${t}_i$. This category encompasses all other cases, typically where ${t}_i$ and ${t}_j$ come from different domains. The length of SRP between ${t}_i$ and ${t}_j$ is defined as infinity, for example, $len\left( srp\left(\mathrm{GO}:0043227\right),e\left(\mathrm{GO}:0044238\right)\right)=+\infty$.

To guide the learning process of GO term embeddings, we introduced the SRP-based partial order constraint: given terms ${t}_i$, ${t}_j$ and ${t}_k$, if ${t}_j$ is closer to ${t}_i$ than ${t}_k$(i.e. $len\left( srp\left({t}_i,{t}_j\right)\right)< len\left( srp\left({t}_i,{t}_k\right)\right)$, then we should have $sim\left(e\left({t}_i\right),e\left({t}_j\right)\right)\succcurlyeq sim\left(e\left({t}_i\right),e\left({t}_k\right)\right)$. For example, $ sim\left(e\left(\mathrm{GO}:0043227\right),e\left(\mathrm{GO}:0043226\right)\right)\succcurlyeq sim\left(e\left(\mathrm{GO}:0043227\right),\right. \left. e\left(\mathrm{GO}:0110165\right)\right)$, $ sim\left(e\left(\mathrm{GO}:0043227\right),e\left(\mathrm{GO}:0043226\right)\right)\succcurlyeq sim\left(e\left(\mathrm{GO}:0043227\right),e\left(\mathrm{GO}:1990900\right)\right)$ and $ sim\left(e\left(\mathrm{GO}:0043227\right),\right. \left.e\left(\mathrm{GO}:0043226\right)\right)\succcurlyeq sim\left(e\left(\mathrm{GO}:0043227\right),e\left(\mathrm{GO}:0044238\right)\right)$ in Figure 1A.

### PO2Vec

We adopt a contrastive learning strategy to learn distinctive embedding of GO terms. Contrastive learning is an unsupervised representation learning method that aims to push positive pairs of ‘similar’ inputs together and push negative pairs of ‘dissimilar’ inputs away in the representation space. The commonly used approach InfoNCE [26] defines the following loss function ${\mathcal{L}}_{InfoNCE}$


(1) \begin{equation*} {\displaystyle \begin{array}{c}{\mathcal{L}}_{InfoNCE}=-\underset{x\in X}{\underbrace{\mathbb{E}}}\left[{\log}\frac{s\left(x,{x}^{+}\right)}{\sum_{x_j\in \mathcal{N}(x)\cup \left\{{x}^{+}\right\}}s\left(x,{x}_j\right)}\right],\end{array}} \end{equation*}

where $X$ contains training examples in a batch, ${x}^{+}$ is the positive sample of $x$ and $\mathcal{N}(x)$ is the set of negative terms of $x$. $s\left(x,{x}_j\right)$ is the similarity between $x$ and ${x}_j$.

In contrastive learning, one of the most important tasks is to sample the positive sample ${t}_i^{+}$ and the negative sample set $\mathcal{N}\left({t}_i\right)$ for the term ${t}_i$. In the implementation of PO2Vec, we exclusively choose positive samples from the terms that are directly reachable with SRP length of 1 (i.e. parent terms or children terms) or indirectly reachable with SRP length of 2.5 (i.e. sibling terms), as positive samples for two main reasons. First, the parent–child and sibling relationships are the closest biological relationships between GO terms. Choosing terms as positive samples based on these relationships aligns with biological intuition. Second, the total number of directly and indirectly reachable terms are numerous. Adding the aforementioned constraints during the selection of positive samples can help address sampling bias and enhance the robustness of the algorithm.

Usually, the number of indirectly reachable and unreachable terms is much larger than directly reachable terms. If we simply draw negative samples from all terms, we may get few directly reachable terms that are important in the GO term embedding learning. Therefore, we propose a new negative term sampling method to obtain better negative samples, which is illustrated in Figure 1B. The new method consists of three phases: indexing, sampling and contrastive learning.

#### Indexing

The indexing phase generates three lists for each term for sampling positive and negative terms. Given a term ${t}_i$, we construct above three lists ${\mathcal{Q}}_{dr}\left({t}_i\right)$, ${\mathcal{Q}}_{ir}\left({t}_i\right)$ and ${\mathcal{Q}}_{ur}\left({t}_i\right)$. ${\mathcal{Q}}_{dr}\left({t}_i\right)$ consists of the directly reachable terms of ${t}_i$, ${\mathcal{Q}}_{ir}\left({t}_i\right)$ consists of the indirectly reachable terms of ${t}_i$ and ${\mathcal{Q}}_{ur}\left({t}_i\right)$ consists of the unreachable terms in the different domain of ${t}_i$. Note that the terms in ${\mathcal{Q}}_{dr}\left({t}_i\right)$ and ${\mathcal{Q}}_{ir}\left({t}_i\right)$ must be sorted in ascending order according to the length of SRP to ${t}_i$.

#### Stratified sampling

In the sampling phase, we sample one positive term and $k$ negative terms from the three lists, i.e. ${\mathcal{Q}}_{dr}\left({t}_i\right)$, ${\mathcal{Q}}_{ir}\left({t}_i\right)$ and ${\mathcal{Q}}_{ur}\left({t}_i\right)$. Specifically, given a term ${t}_i$, we randomly sample a directly reachable term with SRP length of 1 from ${\mathcal{Q}}_{dr}\left({t}_i\right)$ or an indirectly reachable term with SRP length of 2.5 as ${t}_i^{+}$. We take a total of $k$ negative samples as the set of negative samples $\mathcal{N}\left({t}_i\right)$, where $\mathcal{N}\left({t}_i\right)$ = ${\mathcal{N}}_{dr}\left({t}_i\right)\cup{\mathcal{N}}_{ir}\left({t}_i\right)\cup{\mathcal{N}}_{ur}\left({t}_i\right)$. ${\mathcal{N}}_{dr}\left({t}_i\right)$ contains $\min \left(k\times u,\left|{\mathcal{Q}}_{dr}\left({t}_i\right)\right|\right)\left(u\in \left(0,1\right)\right)$ terms sampled from ${\mathcal{Q}}_{dr}\left({t}_i\right)$ whose SRP length is greater than $len\left( srp\left({t}_i,{t}_i^{+}\right)\right)$, ${\mathcal{N}}_{ir}\left({t}_i\right)$ contains $k-\left|{\mathcal{N}}_{dr}\left({t}_i\right)\right|\!\left/ \!2\right.$ terms sampled from ${\mathcal{Q}}_{ir}\left({t}_i\right)$ whose SRP length is greater than $len\left( srp\left({t}_i,{t}_i^{+}\right)\right)$ and ${\mathcal{N}}_{ur}\left({t}_i\right)$ contains $k-\left|{\mathcal{N}}_{dr}\left({t}_i\right)\right|\!\left/ \!2\right.$ terms sampled from ${\mathcal{Q}}_{ur}\left({t}_i\right)$. Here, we need to set two hyperparameters $k$ and $u$, where $k$ determines the total number of negative terms and $u$ determines the number of negative samples in ${\mathcal{N}}_{dr}\left({t}_i\right)$. For detailed information on the experimental settings of hyperparameters $k$ and $u$, please refer to the Supplementary Materials.

#### Contrastive learning

Finally, we define the following balanced InfoNCE loss for the GO term embedding learning:


(2) \begin{equation*} {\displaystyle \begin{array}{c}{\mathcal{L}}_{GO}=-\sum_{i=1}^m\log \frac{sim\left(e\left({t}_i\right),e\left({t}_i^{+}\right)\right)}{sim\left(e\left({t}_i\right),e\left({t}_i^{+}\right)\right)+{\mathcal{C}}_{GO}},\end{array}} \end{equation*}

(3) \begin{equation*} {\displaystyle \begin{array}{c}{\mathcal{C}}_{GO}=\frac{k}{3}\left(\sum_{{\mathcal{N}}_R\in \left\{{\mathcal{N}}_{dr},{\mathcal{N}}_{ir},{\mathcal{N}}_{ur}\right\}}\frac{1}{\left|{\mathcal{N}}_R\left({t}_i\right)\right|}\sum_{t_j\in{\mathcal{N}}_R\left({t}_i\right)} sim\left(e\left({t}_i\right),e\left({t}_j\right)\right)\right),\end{array}} \end{equation*}

(4) \begin{equation*} {\displaystyle \begin{array}{c} sim\left(e\left({t}_i\right),e\left({t}_j\right)\right)=\exp \left(e{\left({t}_i\right)}^{\mathrm{T}}\cdot e\left({t}_j\right)\right)\!\left/ \!\tau \right.,\end{array}} \end{equation*}

where τ is a temperature hyperparameter to adjust the result of $sim\left(\cdot, \cdot \right)$. ${\mathcal{C}}_{GO}$ balances two sets ${\mathcal{N}}_{dr}\left({t}_i\right)$, ${\mathcal{N}}_{ir}\left({t}_i\right)$ and ${\mathcal{N}}_{ur}\left({t}_i\right)$ since $\left|{\mathcal{N}}_{dr}\left({t}_i\right)\right|$ is usually much smaller than $\left|{\mathcal{N}}_{ir}\left({t}_i\right)\right|$ or $\left|{\mathcal{N}}_{ur}\left({t}_i\right)\right|$.

### Joint modeling predictor

**Figure 2:**
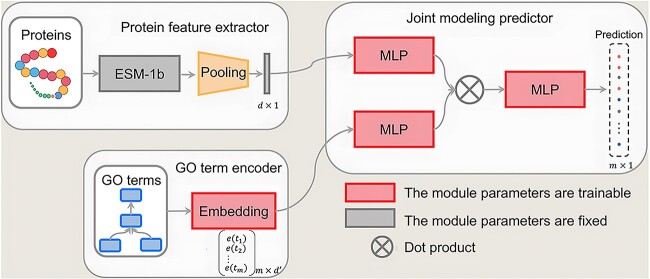
The network framework of PO2GO. The architecture consists of three modules: (i) Protein feature extractor encodes a protein into a $d$ dimension embedding; (ii) GO term encoder encodes each GO term (total $m$ GO terms) into a ${d}^{\prime }$ dimension embedding; and (iii) joint modeling predictor identifies the mapped GO terms for each protein among a total $m$ GO terms.

In our method, we initially derive the GO term embedding using PO2Vec. It’s crucial to emphasize that, during the inference stage, these GO term embeddings remain consistent across different proteins. Essentially, the role of the joint modeling predictor can be interpreted as conducting embedding database searching. We also acquire the protein embedding from the pre-trained ESM-1b. Subsequently, these two kinds of embedding will be used to jointly model protein function prediction. To bridge the gap in the semantic space between GO terms and proteins, we introduce two multi-layer perceptrons (MLPs) to project GO term embeddings and protein embeddings into the same space (Figure 2). Specifically, given a protein ${p}_i$, we obtain protein embedding $f\left({p}_i\right)$ from ESM-1b; then, an MLP is applied to obtain ${f}_{proj}\left({p}_i\right)= MLP\left(f\left({p}_i\right)\right)$. Likewise, given a GO term ${t}_j$, we obtain its projected embedding as ${e}_{proj}\left({t}_j\right)$. We compute the similarity between the protein ${p}_i$ and GO terms by $s\in{\mathbb{R}}^{m\times 1}$, where $m$ is the number of GO terms and the $j$-th element of $s$ is calculated as ${s}_j={e}_{proj}{\left({t}_j\right)}^T\cdot{f}_{proj}\left({p}_i\right)$. Unlike previous works that directly use this similarity vector as the final prediction result, we introduce an MLP layer to obtain the protein function prediction result $\hat{y}\in{\mathbb{R}}^{m\times 1}$, via a multi-label binary cross-entropy loss as follows:


(5) \begin{equation*} {\displaystyle \begin{array}{c}{\mathcal{L}}_{PRED}=-\sum_{j=1}^m\left({y}_j\log{\hat{y}}_j+\left(1-{y}_j\right)\log \left(1-{\hat{y}}_j\right)\right)\end{array}} \end{equation*}

where $y\in{\mathbb{R}}^{m\times 1}\in \left\{0,1\right\}$ denotes whether the GO term ${t}_j$ is annotated to the protein or not.

In the training stage, we fix the protein feature extractor (thus the protein representations are fixed) and learn the parameters of three MLPs by minimizing the loss function in Equation (5) with back propagation. Note that the GO term representations will be tuned in the training stage.

### Benchmark and evaluation

The datasets, metrics and experimental settings for benchmark are detailed in Supplementary Material.

## RESULTS

### Performance evaluation of GO representation learning

We carried out five tasks to thoroughly analyze the learned GO term embeddings in capturing the hierarchical structure in GO, i.e. the depth prediction, distinguishability between ancestor terms and non-ancestor terms, GO domain information encoded by embeddings, correlation with ${sim}_{PFAM}$ and correlation with ${sim}_{PPI}$.

### Depth prediction

The aim of this task is to measure the learned GO term embeddings in distinguishing the depth of GO terms [18]. In this task, we took the GO term embeddings as input and trained a simple MLP to predict the longest path from a given GO term to its root. We randomly split the GO dataset into an 80% training set and a 20% test set.

**Figure 3:**
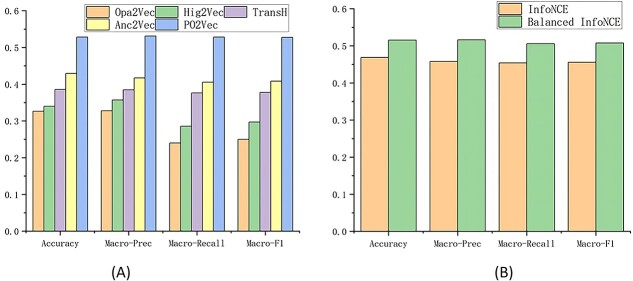
Depth prediction results: (**A**) Comparison with baseline methods and (**B**) PO2Vec with InfoNCE and Balanced InfoNCE.

The depth prediction results of PO2Vec and the baselines are shown in Figure 3A, which indicates that PO2Vec significantly outperforms the baselines in the depth prediction task. This result demonstrates that PO2Vec has superior performance in capturing depth information of a term than baselines.

We conduct an ablation experiment to analyze the benefit of Balanced InfoNCE compared to traditional InfoNCE on capturing depth information. The experimental results are shown in Figure 3B. If we use the default InfoNCE without stratified sampling, the model’s performance in predicting hierarchical depth decreases significantly, resulting from the fact that the directly reachable terms are rarely sampled due to its small size compared to the indirectly reachable and unreachable terms, and thus, the model is undertrained with negative samples dominated by the indirectly reachable and unreachable terms.

### Distinguishability between ancestor terms and non-ancestor terms

**Figure 4:**
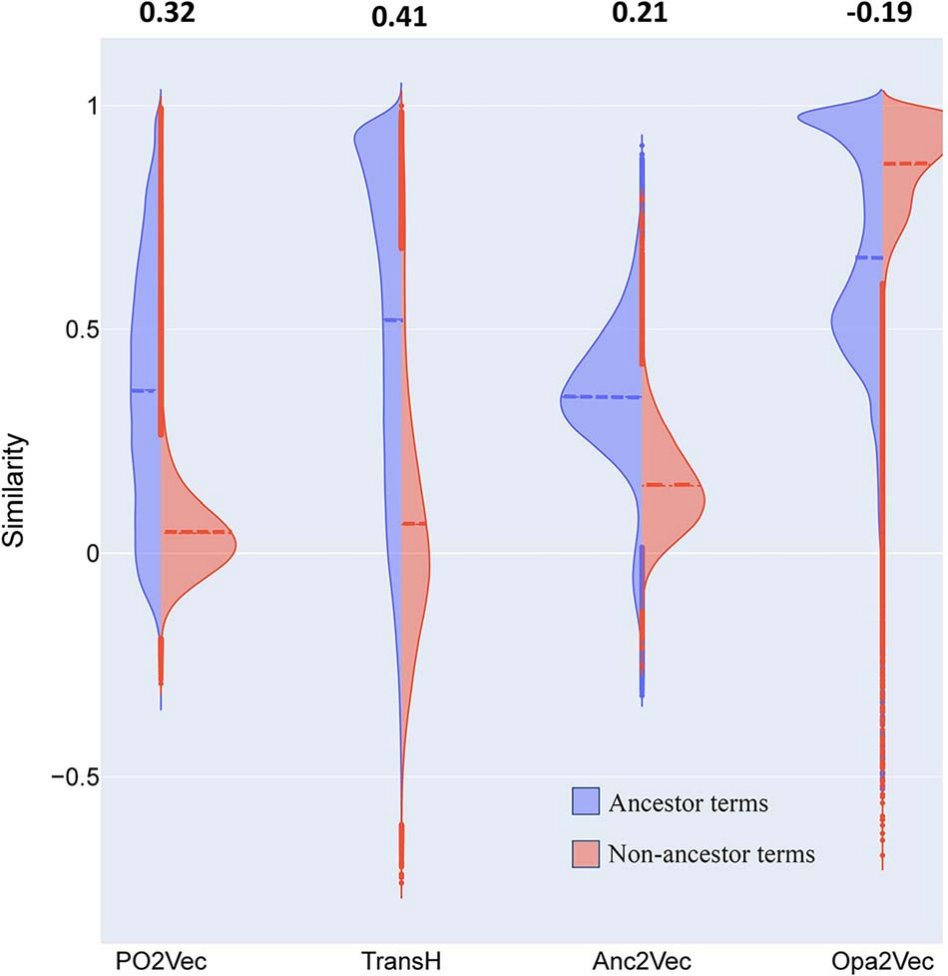
The distributions of cosine similarity of each GO term to its ancestor terms and non-ancestor terms. The 1-Wasserstein distances between the two distributions are shown on the top for each GO embedding method. The negative sign for OPA2Vec indicates that the distance between the ancestor and non-ancestor distributions is in the reverse direction to those of PO2Vec, TransH and Anc2Vec. The dashed lines indicate median of each distribution.

In this task, we calculate the cosine similarity of each GO term to its ancestor terms and non-ancestor terms (Figure 4). Intuitively, a GO term is semantically closer to its ancestors than its non-ancestor terms. The 1-Wasserstein distances [27] computed between the two distributions of similarity for PO2Vec and TransH are larger than Anc2Vec and Opa2Vec (as shown on the top of Figure 4), suggesting that PO2Vec and TransH distinguishes between ancestor terms and non-ancestor terms better than the other methods. The similarity distributions computed from OPA2Vec embeddings show that a given GO term tends to be more similar to its non-ancestor terms than its ancestors, indicating OPA2Vec may not model these two types of GO term relationships very well. The ancestor similarity distribution is more dispersed in PO2Vec than in TransH and Anc2Vec, which implies that PO2Vec is better at resolving the ancestors of varying distances than TransH and Anc2Vec. Taken together, PO2Vec is capable of modeling GO data with hierarchical relationships.

### Domain information encoded by GO term embeddings

**Figure 5:**
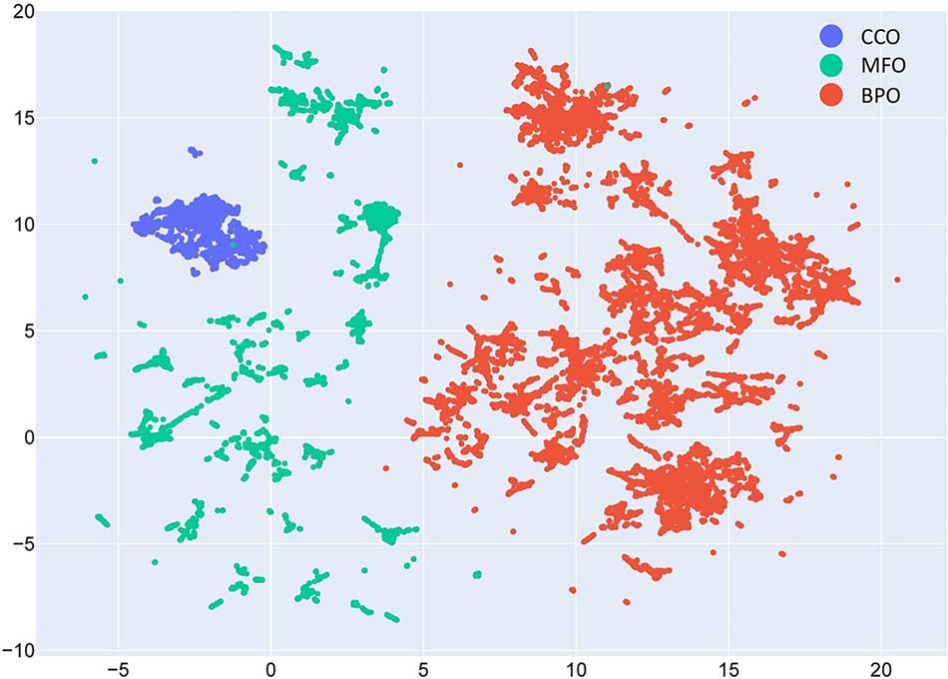
The clustering of GO term embeddings in the three GO domains. Each point represents a GO term.

To evaluate whether the GO domain information is captured in the embeddings for each term, we project the embeddings onto a two-dimensional space with the dimension reduction method Uniform Manifold Approximation and Projection (UMAP) [28] (Figure 5). The result shows a clear separation of three clusters, indicating that PO2Vec is capable of encoding GO terms into proper sub-spaces.

### Correlation with $\boldsymbol{{sim}_{PFAM}}$

The quality of the representation of GO terms can be assessed by comparing protein similarity to their semantic similarity computed from GO term embeddings. The idea is that semantic similarity between the annotated GO terms of two proteins should reflect the similarity of the proteins. Consequently, the correlation between them implicitly indicates the quality of the GO representation. Thus, we use the correlation of semantic similarity with ${sim}_{PFAM}$ to evaluate the performance of PO2Vec and other methods. The semantic similarity between GO annotations of each pair of proteins was computed with the BMA method. Then, the Pearson correlation coefficient and Spearman’s rank correlation coefficient were calculated to evaluate the correlation between the semantic similarity and ${sim}_{PFAM}$.

The results demonstrate that PO2Vec outperforms the other embedding methods in the PFAM-1 and PFAM-3 datasets as well as species divided datasets (Table 1) in most situations. PO2Vec obtained the best performance in all the groups except EC and SC; TransH, which is a strong baseline method for knowledge graph embedding (KGE), yield better results in EC and SC. The performance of PO2Vec in this analysis suggests that its learned GO embeddings may represent protein better than other embedding methods.

**Table 1:** The Pearson correlation coefficient (*p*) and Spearman’s rank correlation coefficient (*s*) between the semantic similarity obtained by different methods against ${sim}_{PPI}$ and ${sim}_{PFAM}$

Dataset	Method	EC		DM		HS		SC		ALL	
		*p*	*s*	*p*	*s*	*p*	*s*	*p*	*s*	*p*	*s*
PFAM-1	Resnik-BMA	0.556	0.595	0.443	0.484	0.693	0.738	0.63	0.667	0.553	0.602
	Seco-BMA	0.55	0.586	0.481	0.505	0.733	0.766	0.601	0.639	0.589	0.63
	Resnik-GIC	0.434	0.56	0.624	0.741	0.648	0.733	0.555	0.665	0.601	0.707
	Seco-GIC	0.455	0.563	0.638	0.742	0.664	0.736	0.569	0.662	0.616	0.708
	OPA2Vec-BMA	0.340	0.429	0.267	0.497	0.371	0.510	0.294	0.447	0.269	0.472
	Hig2Vec-BMA	0.41	0.462	0.681	0.697	0.721	0.747	0.584	0.611	0.647	0.671
	Anc2Vec-BMA	0.458	0.514	0.689	0.746	0.727	0.768	0.587	0.649	0.656	0.708
	TransH-BMA	**0.58**	**0.615**	0.682	0.718	0.716	0.753	**0.66**	**0.691**	0.674	0.71
	PO2Vec-BMA	0.515	0.558	**0.73**	**0.772**	**0.756**	**0.786**	0.645	0.680	**0.699**	**0.737**
PFAM-3	Resnik-BMA	0.534	0.58	0.325	0.371	0.703	0.746	0.627	0.67	0.539	0.591
	Seco-BMA	0.553	0.591	0.329	0.376	0.744	0.775	0.609	0.656	0.574	0.624
	Resnik-GIC	0.428	0.562	0.584	0.711	0.66	0.741	0.534	0.654	0.59	0.7
	Seco-GIC	0.449	0.569	0.599	0.714	0.675	0.742	0.549	0.651	0.605	0.701
	OPA2Vec-BMA	0.287	0.349	0.261	0.508	0.373	0.519	0.284	0.431	0.261	0.468
	Hig2Vec-BMA	0.437	0.484	0.628	0.64	0.73	0.752	0.588	0.624	0.644	0.67
	Anc2Vec-BMA	0.447	0.505	0.649	0.716	0.736	0.775	0.606	0.669	0.653	0.712
	TransH-BMA	**0.577**	**0.624**	0.656	0.688	0.726	0.759	**0.661**	0.692	0.675	0.71
	PO2Vec-BMA	0.509	0.557	**0.699**	**0.742**	**0.764**	**0.793**	0.654	**0.696**	**0.698**	**0.738**
PPI-1	Resnik-BMA	0.699	0.71	**0.858**	0.827	0.489	0.504	0.646	0.65	0.543	0.549
	Seco-BMA	0.709	0.718	0.841	**0.828**	0.505	0.53	0.633	0.644	0.57	0.596
	Resnik-GIC	0.609	0.677	0.717	0.8	0.379	0.477	0.562	0.621	0.466	0.543
	Seco-GIC	0.624	0.688	0.719	0.798	0.389	0.475	0.562	0.617	0.473	0.545
	OPA2Vec-BMA	0.437	0.497	0.623	0.757	0.238	0.352	0.272	0.463	0.232	0.386
	Hig2Vec-BMA	0.665	0.671	0.801	0.813	0.447	0.471	0.529	0.529	0.489	0.503
	Anc2Vec-BMA	0.723	0.738	0.823	0.824	0.482	0.532	0.621	0.645	0.553	0.597
	TransH-BMA	0.66	0.667	0.824	0.814	0.494	0.513	0.603	0.618	0.55	0.571
	PO2Vec-BMA	**0.739**	**0.744**	0.845	0.821	**0.526**	**0.553**	**0.664**	**0.661**	**0.597**	**0.615**
PPI-3	Resnik-BMA	0.674	0.679	**0.805**	0.786	0.493	0.509	0.62	0.632	0.517	0.528
	Seco-BMA	0.689	0.692	0.803	**0.795**	0.509	0.535	0.613	0.627	0.55	0.575
	Resnik-GIC	0.59	0.628	0.688	0.768	0.38	0.481	0.537	0.595	0.438	0.519
	Seco-GIC	0.604	0.641	0.69	0.766	0.39	0.479	0.538	0.59	0.438	0.519
	OPA2Vec-BMA	0.43	0.516	0.555	0.718	0.236	0.357	0.244	0.448	0.208	0.376
	Hig2Vec-BMA	0.673	0.679	0.751	0.778	0.449	0.472	0.492	0.492	0.464	0.48
	Anc2Vec-BMA	0.716	**0.735**	0.779	0.791	0.485	0.535	0.586	0.619	0.523	0.573
	TransH-BMA	0.644	0.648	0.772	0.773	0.498	0.518	0.585	0.6	0.532	0.552
	PO2Vec-BMA	**0.722**	0.724	0.803	0.792	**0.531**	**0.558**	**0.637**	**0.639**	**0.571**	**0.594**

Top performed method in each metric is bold.

### Correlation with $\boldsymbol{{sim}_{PPI}}$

It has been suggested that interacting proteins are likely to be involved in similar subcellular locations or biological processes. As a result, the semantic similarity of two proteins is also a putative predictor of their interaction. Interacting protein pairs should have a higher semantic similarity score than non-interacting protein pairs. Therefore, we evaluate the quality of GO term embeddings by their capability to discriminate proteins as interacting or non-interacting.

Similar to ${sim}_{PFAM}$, we computed the semantic similarity and its Pearson correlation and Spearman’s rank correlation with ${sim}_{PPI}$ (Table 1). The results demonstrate that PO2Vec obtained the highest correlation coefficient in the combined PPI datasets. In the divided protein datasets of species, the performance of PO2Vec is the best (except for Spearman’s correlation in EC) among the GO term embedding methods, closely followed by Anc2Vec.

**Figure 6:**
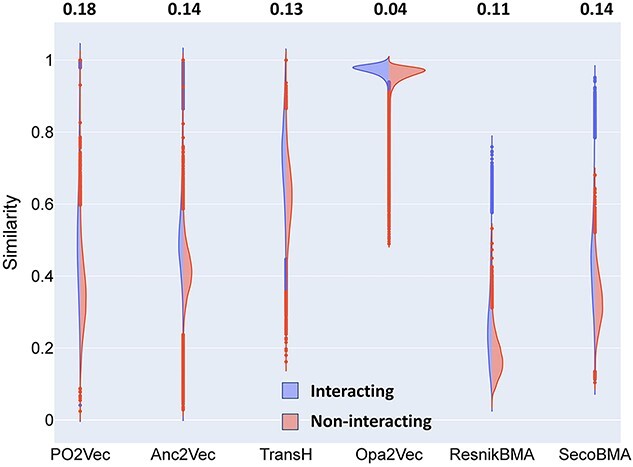
Semantic similarity distributions of the sets of GO terms on PPI datasets. The 1-Wasserstein distances are shown on the top.

For a better comparison, we plotted out the distributions of the semantic similarity for each protein pair, interacting or non-interacting, and computed their 1-Wasserstein distances for each embedding method (Figure 6). The 1-Wasserstein distances obtained from PO2Vec are larger than the others, suggesting that PO2Vec embeddings distinguish interacting proteins from non-interacting ones better. Taken together, these results indicate the superior performance of GO embeddings obtained by PO2Vec in distinguishing whether protein pairs are interacting or not.

### Performance evaluation of protein function prediction

Based on PO2Vec, we developed PO2GO to annotate protein functions with GO and compared its performance to state-of-the-art methods, including TALE, DeepGOA and DeepGOPlus. Similar to PO2GO, both TALE and DeepGOA take advantage of hierarchical information in the GO framework. Note that we replaced the original protein feature extractor of TALE and DeepGOA with ESM-1b for a fair comparison. DeepGOPlus, a classical strong baseline method based on the one-dimensional convolutional neural network, was also included in the benchmark. The performance of the traditional sequence similarity–based method, Diamond, was listed for comparison too. As shown in Table 2, PO2GO outperforms other methods in all three GO domains. The superior performance of PO2GO over TALE and DeepGOA indicates the effectiveness of our PO2Vec embedding method.

**Table 2:** The performance evaluation of different methods for protein function prediction on the CAFA3 dataset and swissprot dataset

Dataset	Method	$\boldsymbol{{F}_{max}}$			$\boldsymbol{{S}_{min}}$			$\boldsymbol{AUPR}$		
		BPO	MFO	CCO	BPO	MFO	CCO	BPO	MFO	CCO
CAFA3	Diamond	0.394	0.542	0.57	23.98	8.86	10.86	0.277	0.463	0.514
	DeepGOPlus	0.451	0.439	0.584	23.57	9.941	11.2	0.354	0.454	0.603
	ESM-1b&DeepGOA	0.431	0.537	0.625	23.33	9.057	10.56	0.379	0.543	0.665
	ESM-1b&TALE	0.471	0.582	0.63	23.79	8.095	10.29	0.381	0.611	0.665
	PO2GO	**0.526**	**0.611**	**0.648**	**21.26**	**7.731**	**9.896**	**0.441**	**0.631**	**0.693**
Swissprot	Diamond	0.486	0.645	0.639	43.665	9.282	13.226	0.362	0.568	0.506
	DeepGOPlus	0.361	0.55	0.612	47.682	12.102	14.213	0.314	0.526	0.646
	ESM-1b&DeepGOA	0.393	0.586	0.639	46.599	11.469	13.474	0.357	0.608	0.689
	ESM-1b&TALE	0.446	0.668	0.673	43.708	9.683	12.343	0.423	0.714	0.736
	PO2GO	**0.514**	**0.697**	**0.719**	**41.477**	**8.437**	**11.82**	**0.457**	**0.753**	**0.752**

Top performed method in each metric is bold.

### Ablation study of PO2GO

To further analyze the contribution of PO2Vec to protein function prediction, we conducted an ablation experiment. When we removed PO2Vec or replaced PO2Vec with a naive multi-hot embedding that could capture the information about GO terms and their ancestors, the performance of PO2GO appeared to degrade (Table 4). These ablation results demonstrate that the GO information captured by PO2Vec helps to enhance the predictive performance of the model for protein annotation. We also found that the prediction performance of PO2GO did not decrease significantly when we fixed the pre-trained PO2Vec embeddings during PO2GO training. This shows that the embedding pre-training procedure of PO2Vec does learn good GO term representations suitable for downstream tasks.

### The specificity of protein annotation

To explore characteristics of protein annotation by PO2GO, we compare PO2GO with competing methods in terms of specificity as defined by information content (IC) [19, 29]. A term with high IC tends to be more specialized and rarer in occurrence, and a term with low IC tends to have a broad function and appears more commonly in protein annotations. We calculate the average IC of true-positive predictions of a protein for four different methods at their best threshold (calculated by ${F}_{max}$), respectively. For detailed description, please refer to the Supplementary Material. In BPO and MFO, PO2GO obtains the best average IC and significantly outperforms the other competing methods (Table 3). In CCO, TALE slightly outperforms PO2GO and achieves the best average IC. Different from MFO and BPO, the average depth of GO terms is shallower in CCO, which is 6.2 in CAFA3 (as compared to 6.8 and 8.3 in MFO and BPO, respectively). Therefore, our PO2Vec embedding approach probably models deeper GO hierarchical structure better, which consequently results in better performance in MFO and BPO than in CCO. In summary, PO2GO tends to generate higher or comparable specificity predictions for protein annotation.

**Table 3:** Average information content of the predictions on CAFA3 dataset

Method	MFO	BPO	CCO
DeepGOPlus	4.07	6.56	2.52
DeepGOA	4.00	7.01	3.26
TALE	5.28	8.16	**3.90**
PO2GO	**5.78**	**9.46**	3.76

Top performed method in each metric is bold.

**Table 4:** AUPR of the predictions on CAFA3 dataset in the ablation analysis

Method	MFO	BPO	CCO
PO2GO w/o PO2Vec	0.627	0.423	0.686
PO2GO w/ multi-hot embeddings	0.62	0.43	0.688
PO2GO w/ fixed PO2Vec	0.627	0.437	**0.693**
PO2GO	**0.631**	**0.441**	**0.693**

Top performed method in each metric is bold.

### Few-shot prediction

**Figure 7:**
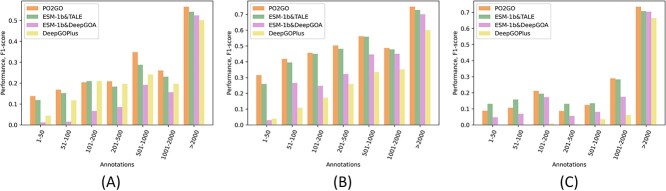
The term-centric F1-score evaluation is grouped by annotation size on the CAFA3 dataset. (**A**) BPO evaluation; (**B**) MFO evaluation; and (**C**) CCO evaluation.

There are protein families with few numbers of known sequences; thus, it is important to assess how the protein annotation models perform when the training examples are insufficient. For this purpose, we group GO terms according to their numbers of annotated proteins and calculate the GO term-centric F1-score within each group as an evaluation metric when the best threshold (calculated by ${F}_{max}$) is selected. As shown in Figure 7, the performances of all four methods deteriorate noticeably as the available proteins decrease, indicating that current deep learning models usually need sufficient training examples to lead to good protein annotation prediction. Compared with the other methods, this performance deterioration is mitigated in PO2GO, suggesting that high-quality GO term embedding pre-training can compensate for the shortage of training examples to some extent. The PO2GO performance in CCO (Figure 7C) is not consistently better than the other methods as in BPO and MFO, probably due to the similar phenomenon of CCO’s shallow hierarchical structure as observed in the annotation specificity analysis. Overall, PO2GO has better predictive performance in the majority of scenarios.

## CONCLUSION AND DISCUSSION

A novel model named PO2Vec is presented for GO term representation learning. Under the SRP-based partial order constraint, the ancestral or non-ancestral properties of terms were captured by the contrastive learning, resembling the learning process in previous co-occurrence-based methods. We included terms across GO domains for similarity computation so that all the terms from the three domains can be mapped to the same embedding space, although this may have limited direct biological implications. In this process, we assumed that the similarity between terms from different domains is smaller than that of any term pairs with ancestral relationship. PO2Vec further refines the learned ancestral information to distinguish proximities of terms with distant ancestors and close ancestors. Overall, PO2Vec captures the GO topological information more comprehensively under the SRP-based partial order constraint. The effectiveness of PO2Vec was demonstrated in experimental analyses from five aspects on both GO and protein levels. On the GO level experiments, we demonstrate that the learned embedding through PO2Vec can well reconstruct term depth, differentiate ancestor and non-ancestor GO terms and identify their own GO domains. In addition, on the protein level, we find that the embedding of PO2Vec reflects the functional domain and interacting of proteins, indicating that our embedding is suitable for a wide range of gene-related bioinformatics analyses.

A novel protein function annotation model, called PO2GO, is proposed to integrate PO2Vec and the protein language pre-trained model ESM-1b. The effectiveness of PO2GO is demonstrated with benchmarks by comparing to DeepGOA, TALE and DeepGOPlus as well as Diamond. In comparison to simpler methods such as graph neural network and graph-based regularization techniques, PO2Vec provides a deeper semantic representation by effectively distinguishing between varying degrees of term proximities through a contrastive learning framework. This suggests that the high-quality GO term embedding helps improve computational protein annotation. Consequently, GO term embedding not only improves the overall performance of protein function prediction as measured in the metrics of ${F}_{max}$, ${S}_{min}$ and AUPR but also enhances the specificity of prediction and the few-shot ability of the model. The enhancement of prediction specificity can facilitate the annotation of a protein reliably with specific GO terms rather than generic, shallow-level ones. The improvement of the model’s few-shot ability provides a feasible solution for biological tasks, including protein function prediction, which are hindered by limited availability of labeled data. The ESM-1b we adopted in the PO2GO architecture can be replaced by other well-trained protein encoders since our formulation is generic enough that any pre-trained protein language model may be used. Moreover, our embedding approach may also provide insight for the representation learning of other knowledge graphs such as the Human Phenotype Ontology [30], Disease Ontology [31] and SNOMED CT [32].

One limitation of this study is that the SRP-based partial order constraint takes into account only is_a and part_of relationships in the GO system. Although these two types of relationships make up over 88% of all the GO term relationships, the embedding could potentially be further improved if the rest relationships are included in the representation learning. Moreover, the textual description of each GO term is another source of information that could be processed by NLP models in addition to the relationship information in the GO DAGs, which may also enhance the representation learning of GO terms.

**Key Points**

- A novel model named PO2Vec is presented for Gene Ontology (GO) term representation learning.
- Compared with existing methods based on GO-directed acyclic graph structure, PO2Vec captures the topological information of GO more comprehensively under the shortest reachable path–based partial order constraint. The effectiveness of PO2Vec was demonstrated in experimental analyses from five aspects.
- A novel protein function annotation prediction model, named PO2GO, is proposed, which is jointly constructed by PO2Vec and the protein language pre-trained model ESM-1b. The superior performance of PO2GO is demonstrated with comparative benchmarks.

## Supplementary Material

revised-supplementary_non-marked-up-version_bbae077

## Data Availability

Source code of PO2Vec and PO2GO is available at https://github.com/xbiome/protein-annotation.

## Author Biographies

**Wenjing Li** is currently a M student at College of Computer Science and Software, Shenzhen University. His research interests include deep learning and bioinformatics.

**Bin Wang** is currently a PhD student at School of Mathematics and Computer Sciences, Nanchang University. His research research focuses on natural language processing and machine learning for bioinformatics.

**Jin Dai** is an associate professor in the School of Physics at Beijing Institute of Technology. His research interests include protein dynamics, and deep learning methods for protein modelling.

**Yan Kou**, based in New York, NY, US, is currently a Sr. Director, Onyx Product Management at GSK, bringing experience from previous roles at Xbiome and Insight Data Science. Yan Kou holds a 2010–2015 Doctor of Philosophy (Ph.D.) in Bioinformatics at Icahn School of Medicine at Mount Sinai.

**Xiaojun Chen** is an associate professor at College of Computer Science and Software, Shenzhen University. His research interests include clustering, graph mining, text mining and distributed machine learning.

**Yi Pan** is a Chair Professor and the Dean of the College of Computer Science and Control Engineering, Shenzhen Institute of Advanced Technology, Chinese Academy of Sciences, China, and a Regents' Professor Emeritus with Georgia State University, USA. His current research interests include bioinformatics and health informatics using big data analytics, cloud computing, and machine learning technologies.

**Shuangwei Hu**, basedin Hangzhou, China, is currently a Sr. AI Director at Xbiome, leveraging extensive experience from the internet industry and biophysics research. Shuangwei Hu earned a Ph.D. degree in Physics from Uppsala University in Sweden during 2008–2012.

**Zhenjiang Zech Xu** is a professor at State Key Laboratory of Food Science and Technology, Nanchang University. His research interests include bioinformatics, statistics & machine learning, and microbiome.
