# Supplementary material for: Partial order relation–based gene ontology embedding improves protein function prediction
Source: Brief Bioinform. 2024 Mar 5;25(2):bbae077. doi: 10.1093/bib/bbae077 (PMC10917077; doi:10.1093/bib/bbae077)
Supplement: revised-supplementary_non-marked-up-version_bbae077 [file revised-supplementary_non-marked-up-version_bbae077.docx]

# Supplementary material of “PO2Vec: partial order relation-based gene ontology embedding improves protein function prediction”

## Datasets

Table S1. Statistics of GO relationships in the GO dataset

| Relationship | Count | Proportion |
| --- | --- | --- |
| is_a | 71846 | 79.2% |
| part_of | 8149 | 9.0% |
| has_part | 802 | 0.8% |
| regulates | 3552 | 3.9% |
| negatively_regulates | 3085 | 3.4% |
| positively_regulates | 3065 | 3.3% |
| occurs_in | 192 | 0.2% |

**Gene ontology.** We used the GO data which was published on 2019-10-07, removed the isolated terms, replaced the obsolete terms ID with their primary ID, and retained the relationships is_a and part_of only. Finally, we obtain 29,457 terms in the BPO domain, 11,093 terms in the MFO domain, and 4,183 terms in the CCO domain.

**GO representation benchmark datasets**. To evaluate the performance of PO2Vec and other baseline methods, we conduct the benchmark using datasets proposed by [1], including the PPI dataset and PFAM dataset. The information about included proteins and protein pairs of the PPI dataset and PFAM dataset is presented in Table S2 and Table S3. The PPI and PFAM datasets contain proteins of four species: D. melanogaster (DM), E. Coli (EC), H. sapiens (HS), S. cerevisiae (SC), and combined all four species (ALL). In datasets with a suffix ending in ”1”, proteins must have at least one annotation in each GO domain and there should be at least one leaf annotation in at least one GO domain. In datasets with a suffix ending in ”3”, proteins must have at least one annotation in each GO domain and there should be at least one leaf annotation in each GO domain. More details are elaborated in the study [1].

Following [1], two proxies for protein similarity based on their biological properties were employed to measure the similarity between two proteins. In the PPI dataset, the proxy is protein-protein interaction ${sim}_{PPI}$ which has a binary representation: 1 if the proteins interact, 0 otherwise. Two proteins are considered to be similar if they interact. In the PFAM dataset, the proxy is ${sim}_{PFAM}$ which measures the number of overlapping protein domains between each pair of protein sequences [2].

Table S2. Statistical information on the PPI benchmark datasets

| Datasets | Species | Proteins | Pairs |
| --- | --- | --- | --- |
| DM-PPI-1 | D. melanogaster | 455 | 364 |
| EC-PPI-1 | E. coli | 371 | 734 |
| HS-PPI-1 | H. sapiens | 7093 | 30826 |
| SC-PPI-1 | S. cerevisiae | 3776 | 27898 |
| ALL-PPI-1 | ALL | 11695 | 59822 |
| DM-PPI-3 | D. melanogaster | 287 | 200 |
| EC-PPI-3 | E. coli | 263 | 420 |
| HS-PPI-3 | H. sapiens | 6781 | 29672 |
| SC-PPI-3 | S. cerevisiae | 2888 | 16904 |
| ALL-PPI-3 | ALL | 10156 | 47196 |

Table S3. Statistical information on the PFAM benchmark datasets

| Datasets | Species | Proteins | Pairs |
| --- | --- | --- | --- |
| DM-PFAM-1 | D. melanogaster | 7470 | 31350 |
| EC-PFAM-1 | E. coli | 1231 | 3363 |
| HS-PFAM-1 | H. sapiens | 13246 | 31350 |
| SC-PFAM-1 | S. cerevisiae | 4782 | 38166 |
| ALL-PFAM-1 | ALL | 26729 | 104229 |
| DM-PFAM-3 | D. melanogaster | 5300 | 17682 |
| EC-PFAM-3 | E. coli | 724 | 1332 |
| HS-PFAM-3 | H. sapiens | 11666 | 25527 |
| SC-PFAM-3 | S. cerevisiae | 3660 | 29265 |
| ALL-PFAM-3 | ALL | 21350 | 73806 |

**Protein annotation benchmark datasets**. To evaluate our PO2GO approach, We utilized two datasets: the CAFA3 dataset and the SwissProt dataset. The CAFA3 dataset was derived from an international protein function prediction competition. In this dataset, experimental functional annotations are used as functional annotations of proteins [3]. It contains 50,813 training samples and 2,133 test samples for the BPO domain, 35,086 training samples and 1088 test samples for the MFO domain, 49,328 training samples and 1,094 test samples for the CCO domain (see Table S4).

For the SwissProt dataset, followed by [4], we downloaded SwissProt dataset in Uniprot website (release 2023_05) and divided proteins into training and testing sets based on sequence identity. Specifically, proteins over 50% sequence identity were grouped together, with 90% of these groups allocated for training and the remaining 10% for testing. The SwissProt dataset used in our study contains 49,003 training samples and 5,402 test samples for the BPO domain, 36,403 training samples and 4,038 test samples for the MFO domain, and 47,177 training samples and 5,165 test samples for the CCO domain. These sequences are annotated with a total of 19,832 GO terms for BPO, 6,785 for MFO, and 2,760 for CCO (refer to Table S4).

We assessed the performance of PO2GO using Fmax, Smin, and AUPR metrics for both CAFA3 and Swissprot datasets. Additionally, an ablation study, along with analyses of specificity and the few-shot ability of PO2GO were conducted exclusively on the CAFA3 dataset.

Table S4. Summary of protein annotation benchmark datasets

|  | Statistics | BPO | MFO | CCO |
| --- | --- | --- | --- | --- |
|  | Seq in Training Set | 50813 | 35086 | 49328 |
| CAFA3 | Seq in Testing Set | 2133 | 1088 | 1094 |
|  | Number of GO terms | 19901 | 6367 | 2470 |
|  | Seq in Training Set | 49003 | 36403 | 47177 |
| Swissprot | Seq in Testing Set | 5402 | 4038 | 5165 |
|  | Number of GO terms | 19832 | 6785 | 2760 |

## Experimental settings

**Metrics.** We adopted evaluation metrics reported in the previous studies [1,5–7], including the Pearson correlation coefficient, Spearman’s rank correlation coefficient, accuracy, macro-precision, macro-recall, and macro-F1 score, to measure the quality of learned GO term embeddings, where the macro average calculates the performance of each class and then calculates their average. Following the study [8], we also use $F_{max}$, $S_{min}$ and the area under precision-recall curve (AUPR) to evaluate the performance of protein function annotation tasks, where $F_{max}$ is the maximum F1-score in which the threshold for prediction was set to $\{0, 0.01, \cdot\cdot\cdot, 0.99, 1\}$. $S_{min}$ indicates the semantic distance between predicted and actual annotations. AUPR is the area of the region enclosed by the precision-recall curve and the coordinate axes.

**Baselines.** In the GO term embedding task, we chose OPA2Vec[9], Hig2Vec [5], Anc2Vec [10] and TransH[10] as baseline methods for comparison, where the hyper-parameters of each baseline were set the same as the original papers.

In the protein function annotation task, we compared our proposed method with TALE [11], DeepGOA [12], and DeepGOPlus [8], in which we replaced the protein feature extractors of DeepGOA and TALE with ESM-1b (fixed parameters) for a fair comparison. Similarly, we used the hyper-parameters in the original paper in our experiment.

**Implementation details.** Following the previous studies [6,10], we used the best match average (BMA) [13] (Eq. S1) method to compute the similarity between two sets of GO terms using their term embeddings. Then the embedding quality can be assessed by the correlation of the BMA score against the protein similarity proxies (${sim}_{PPI}$ and ${sim}_{PFAM}$) for each pair of proteins. For the purpose of comparison, we also computed BMA and GIC scores using the Resnik and Seco information content (IC) [14]. GIC [15] (Eq. S2) is another groupwise approach that adopts the Jaccard similarity.
$\begin{aligned} \mathrm{BMA}\left( \mathcal{T}_{i},\mathcal{T}_{j} \right)=\frac{1}{2}\left( \frac{1}{\left| \mathcal{T}_{i} \right|}\sum_{t_{1}\in\mathcal{T}_{i}} \max_{t_{2}\in\mathcal{T}_{j}} s\left( t_{1},t_{2} \right)+\frac{1}{\left| \mathcal{T}_{j} \right|}\sum_{t_{2}\in\mathcal{T}_{j}} \max_{t_{1}\in\mathcal{T}_{i}} s\left( t_{1},t_{2} \right) \right),\#\left( S1 \right) \end{aligned}$

where $\mathcal{T}_{i}$ and $\mathcal{T}_{j}$ denote two sets of protein function annotations, respectively, and $s\left( t_{1},t_{2} \right)$ is the similarity between $t_{1}$ and $t_{2}$. The similarity is cosine similarity of ${e(t}_{1})$ and ${e(t}_{2})$ in the embedding methods (OPA2Vec, Hig2Vec, Anc2Vec, TransH, and PO2Vec); in IC-related methods (Resnik, Seco), the similarity is calculated as $s\left( t_{1},t_{2} \right)=\max\left[ IC\left( t \right) \right] :t\in A\left( t_{1} \right)\cap A\left( t_{2} \right)$, where$IC\left( t \right)$ is the IC value of $t$ and $A\left( t_{1} \right)$ is the set of ancestors of $t_{1}$.

$$\begin{aligned} \mathrm{GIC}\left( \mathcal{T}_{i},\mathcal{T}_{j} \right)=\frac{\sum_{t\in\mathcal{T}_{i}\cap\mathcal{T}_{j}} IC\left( t \right)}{\sum_{t\in\mathcal{T}_{i}\cup\mathcal{T}_{j}} IC\left( t \right)},\#\left( S2 \right) \end{aligned}$$

where $\mathcal{T}_{i}$ and $\mathcal{T}_{j}$ denote two sets of protein function annotations, respectively, and $IC\left( t \right)$ is the IC value of GO term $t$.

We ran PO2Vec 400 epochs with a batch size of 3000 to obtain the GO term embedding, where the temperature parameter τ in Eq. (4) was set to 0.1 by default. We used the Adam optimizer with a learning rate of 5e-2 to train the model.For training PO2GO, the epoch number was set to 30 and the batch size was set to 32; the optimizer was Adam with a learning rate of 1e-4, and the weight decay was set to 0.1.

**Hyperparameters optimization.** In contrastive learning, one of the most important tasks is to sample the positive sample and negative samples. We defined two hyperparameters to control the negative sampling process and their values are optimized based on the following rules. The hyperparameter “k” represents the total number of negative samples. We conducted a hyperparameter search for k, using the same evaluation criterion as the "Distinguishability between ancestor terms and non-ancestor terms" subsection in the “Result” section of the manuscript. In this evaluation, we computed the distribution divergence of the cosine similarities between each GO term and its ancestor terms and non-ancestor terms for different k values. We found that the model is insensitive to its value (Fig. S1.). Ultimately, we opted for a relatively large value (k=100) to include more samples for model training. The hyperparameter “u” corresponds to the proportion of negative samples from ancestor terms, which is empirically set to 0.2 for two main reasons: Firstly, the number of ancestors available for sampling should be larger than u*k. Secondly, this particular setting minimizes the divergence between the sampled ancestor terms and the non-ancestor terms for optimal contrastive learning efficacy. To summarize, we have determined the hyperparameter settings to be k=100 and u=0.2.


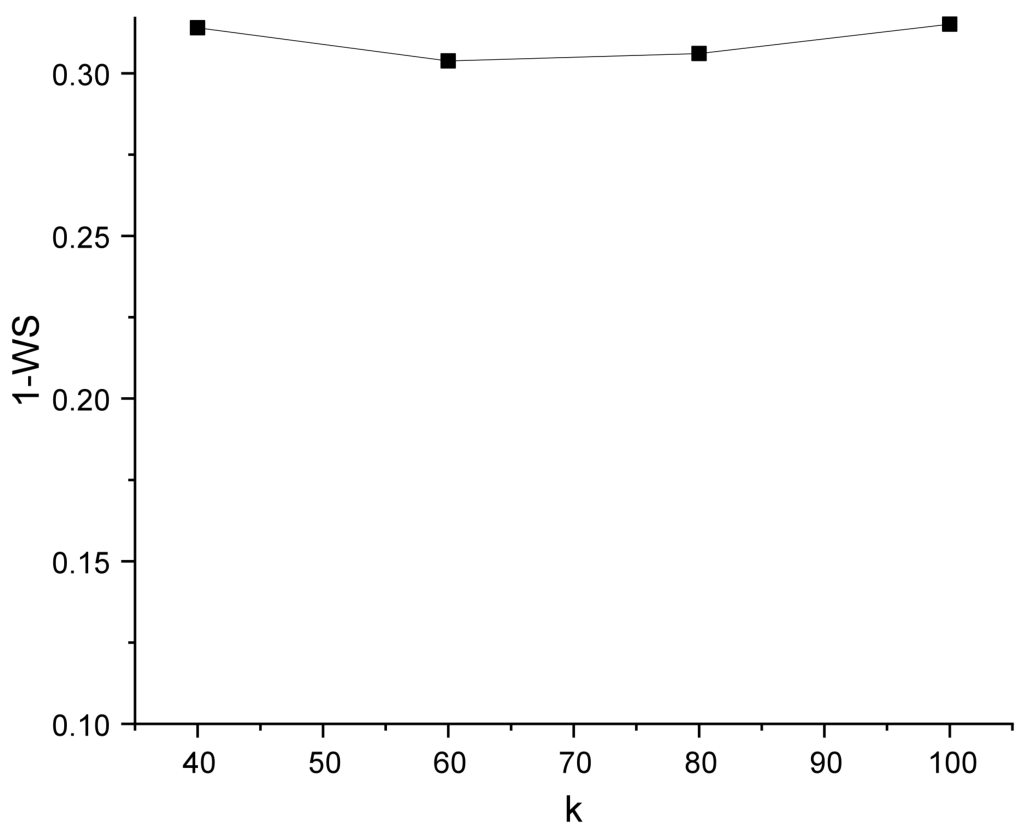


Fig. S1. The optimization process of hyperparameter k. The 1-Wasserstein distances between distributions of simalities of ancestor pairs and non-ancestor pairs for values of k ranging from 40 to 100.

**IC-based specificity.** We computed Information Content (IC) of GO term predictions similarly to DeepGOZero [4]. Briefly, we converted the probability predictions of each method into hard labels {0,1}, using the optimal threshold t yielded from the calculation of $F_{max}$. Then, we calculate the average IC value for true positive predicted GO terms, $average\_IC=\frac{1}{n}\sum_{j=1}^{n} \frac{1}{m}\sum_{i=1}^{m} IC(c_{i})$, where $n$ is the total number of protein in test dataset, $m$ is the number of terms whose predicted probability are greater than threshold t, and $IC(c)= -\log[Pr(c|P(c))]$, where $P(c)$ is a set of parent classes of the GO term $c$ and $Pr(\cdot)$ is the probability.

## References

1. Cardoso C, Sousa RT, Köhler S, et al. A Collection of Benchmark Data Sets for Knowledge Graph-based Similarity in the Biomedical Domain. Database 2020;**2020**.

2. El-Gebali S, Mistry J, Bateman A, et al. The Pfam protein families database in 2019. Nucleic Acids Research 2019; **47**:D427–D432

3. Zhou N, Jiang Y, Bergquist TR, et al. The CAFA challenge reports improved protein function prediction and new functional annotations for hundreds of genes through experimental screens. Genome Biology 2019; **20**:244

4. Kulmanov M, Hoehndorf R. DeepGOZero: improving protein function prediction from sequence and zero-shot learning based on ontology axioms. Bioinformatics 2022; **38**:i238–i245

5. Kim J, Kim D, Sohn K-A. HiG2Vec: hierarchical representations of Gene Ontology and genes in the Poincaré ball. Bioinformatics 2021; 37:2971–2980

6. Zhao L, Sun H, Cao X, et al. Learning representations for gene ontology terms by jointly encoding graph structure and textual node descriptors. Briefings in Bioinformatics 2022; **23**:bbac318

7. Grandini M, Bagli E, Visani G. Metrics for multi-class classification: an overview. arXiv preprint arXiv:2008.05756 2020;

8. Kulmanov M, Hoehndorf R. DeepGOPlus: improved protein function prediction from sequence. Bioinformatics 2019; **36**(2):422-429.

9. Smaili FZ, Gao X, Hoehndorf R. OPA2Vec: combining formal and informal content of biomedical ontologies to improve similarity-based prediction. Bioinformatics 2019; **35**:2133–2140

10. Edera AA, Milone DH, Stegmayer G. Anc2vec: embedding gene ontology terms by preserving ancestors relationships. Briefings in Bioinformatics 2022; **23**:bbac003

11. Cao Y, Shen Y. TALE: Transformer-based protein function Annotation with joint sequence–Label Embedding. 2021; **37**(18): 2825-2833.

12. Zhou G, Wang J, Zhang X, et al. DeepGOA: Predicting Gene Ontology Annotations of Proteins via Graph Convolutional Network. 2019 IEEE International Conference on Bioinformatics and Biomedicine (BIBM) 2019; 1836–1841

13. Azuaje F, Wang H, Bodenreider O. Ontology-driven similarity approaches to supporting gene functional assessment. Proceedings of the ISMB’2005 SIG meeting on Bio-ontologies 2005; **2005**:9–10

14. Guzzi PH, Mina M, Guerra C, et al. Semantic similarity analysis of protein data: assessment with biological features and issues. Briefings in Bioinformatics 2012; **13**:569–585

15. Pesquita C, Faria D, Bastos H, et al. Metrics for GO based protein semantic similarity: a systematic evaluation. BMC Bioinformatics 2008; **9**:S4
